# Decreased Exercise-Induced Changes in Prefrontal Cortex Hemodynamics Are Associated With Depressive Symptoms

**DOI:** 10.3389/fnrgo.2022.806485

**Published:** 2022-05-20

**Authors:** James Crum, Flaminia Ronca, George Herbert, Sabina Funk, Estela Carmona, Uzair Hakim, Isla Jones, Mark Hamer, Joy Hirsch, Antonia Hamilton, Ilias Tachtsidis, Paul W. Burgess

**Affiliations:** ^1^Institute of Cognitive Neuroscience, Faculty of Brain Sciences, University College London, London, United Kingdom; ^2^Institute of Sport Exercise and Health, Faculty of Medical Sciences, University College London, London, United Kingdom; ^3^Department of Medical Physics and Biomedical Engineering, Faculty of Engineering Sciences, University College London, London, United Kingdom; ^4^Department of Comparative Medicine, School of Medicine, Yale University, New Haven, CT, United States; ^5^Department of Psychiatry, School of Medicine, Yale University, New Haven, CT, United States; ^6^Department of Neuroscience, School of Medicine, Yale University, New Haven, CT, United States

**Keywords:** depression, aerobic fitness (VO_2max_), exercise neuroscience, fNIRS (functional near infrared spectroscopy), frontal lobe, executive functions

## Abstract

People with a depressed mood tend to perform poorly on executive function tasks, which require much of the prefrontal cortex (PFC), an area of the brain which has also been shown to be hypo-active in this population. Recent research has suggested that these aspects of cognition might be improved through physical activity and cognitive training. However, whether the acute effects of exercise on PFC activation during executive function tasks vary with depressive symptoms remains unclear. To investigate these effects, 106 participants were given a cardiopulmonary exercise test (CPET) and were administered a set of executive function tests directly before and after the CPET assessment. The composite effects of exercise on the PFC (all experimental blocks) showed bilateral activation changes in dorsolateral (BA46/9) and ventrolateral (BA44/45) PFC, with the greatest changes occurring in rostral PFC (BA10). The effects observed in right ventrolateral PFC varied depending on level of depressive symptoms (13% variance explained); the changes in activation were less for higher levels. There was also a positive relationship between CPET scores (VO_2_peak) and right rostral PFC, in that greater activation changes in right BA10 were predictive of higher levels of aerobic fitness (9% variance explained). Since acute exercise ipsilaterally affected this PFC subregion and the inferior frontal gyrus during executive function tasks, this suggests physical activity might benefit the executive functions these subregions support. And because physical fitness and depressive symptoms explained some degree of cerebral upregulation to these subregions, physical activity might more specifically facilitate the engagement of executive functions that are typically associated with hypoactivation in depressed populations. Future research might investigate this possibility in clinical populations, particularly the neural effects of physical activity used in combination with mental health interventions.

## Introduction

There appears to be a link between performance on executive function tasks and psychopathological symptomology (see Joormann and Vanderlind, 2014; Rock et al., 2014, for reviews). For example, in the case of depression, greater symptom severity and maladaptive strategies for downregulating negative emotion are associated with worse performance on tasks requiring response inhibition (e.g., Joormann and Gotlib, 2010; Joormann et al., 2011), dynamic updating (e.g., Meiran et al., 2011), and attentional switching (e.g., Malooly et al., 2013). The most prevalent maladaptive strategy is rumination, a trait-like proclivity to think repetitively about goal-incongruent and mood-congruent information (Nolen-Hoeksema, 1991; Nolen-Hoeksema et al., 2008). Whether rumination and similar deleterious tendencies (e.g., forms of attentional suppression; Campbell-Sills et al., 2006) are integral to the pathogenesis of psychopathological symptoms (Wells and Matthews, 2014) or a product of them, such propensities are nonetheless concurrent with diminished mental health, and present a challenge to treatment. One reason why maladaptive emotion regulation strategies impede the cultivation of mental health might be that they place particular demands on executive functions, leaving fewer cognitive resources available for engaging in adaptive strategies that also require executive functions, such as reappraisal (see Joormann and Siemer, 2014, for review). The clinical implication is that deficits in executive functions might limit the effectiveness of mental health interventions such as psychotherapy (Roiser and Sahakian, 2013). This is because the neurocognitive mechanisms potentially mediating the effects of non-pharmacological interventions (e.g., recogitation; Crum, 2021a,b), which also likely drive explicit, conscious cognitive strategies to downregulate negative emotion (see Braunstein et al., 2017, for review), are predominately executive in nature. This raises the interesting question of whether improving performance of the executive operations localized within the prefrontal cortex (PFC; see Knight and Stuss, 2013; Shallice and Cooper, for reviews) might augment the effects of these mechanisms on mental health.

Meta-analyses of studies aiming to improve cognition in this way (e.g., attention bias modification) generally support their effectiveness in reducing symptoms common to anxiety and mood disorders (e.g., Hakamata et al., 2010; Beard et al., 2012; Cristea et al., 2015; Liu et al., 2017; see Siegle et al., 2007; Keshavan et al., 2014, for reviews). Such paradigms target executive functions directly and, therefore, adopt a “top-down” approach to improving mental health. There is also some evidence for the effectiveness of transcranial direct current stimulation in reducing depressive symptoms (e.g., Katz et al., 2017; Ruf et al., 2017), which represents a more “bottom-up” approach. However, the findings of cognitive training paradigms are often mixed in the sense that there are marked individual differences within samples; training works differently for different people. Accounting for these differences likely requires training programs to be customized to the individual rather than to a particular group. Moreover, such interventions have not yet reached the point at which they are practical for people to do in their everyday lives. Thus, there is a growing interest in whether cognitive functions can be improved through top-down training approaches, and although the evidence crossing into the clinical domain is promising, it is still marginal and further research is necessary. Such approaches should only be considered as a complement rather than a replacement to mental health interventions that have long been established as efficacious (e.g., McArdle et al., 2012).

It is plausible that some bottom-up approaches to improving cognition might be more practical and able to circumvent the challenges of targeting specific cognitive functions in the brain. For example, one potential approach is medication; however, evidence for the effectiveness of medication in improving cognition in clinical populations is not strong (Halahakoon and Roiser, 2016; Shilyansky et al., 2016). Another possibility is one of the most predominant means by which to improve wellbeing in everyday life: physical activity. Physical activity combined with cognitive training appears to benefit brain function (e.g., Zhu et al., 2016). These improvements to “cold” cognition might benefit psychological wellbeing in turn. Indeed, habitual exercise which places cardiorespiratory demands on the body (e.g., running, long walks, strength training, yoga, etc.) has been associated with mental health improvements (Teychenne et al., 2008; Harvey et al., 2010; Song et al., 2012; Mammen and Faulkner, 2013; Hallgren et al., 2016), and it appears that both low and high levels of cardiorespiratory intensity work to engender mood changes (Helgadóttir et al., 2016). Unsurprisingly, then, exercise has been explored as an alternative treatment for mild to moderate depression (Parker and Crawford, 2007), as well as other disorders (see Stathopoulou et al., 2006). More appropriately, using exercise in combination with cognitive–behavioral therapy (CBT) has shown improvements in outcome measures relative to CBT with no exercise program (e.g., McArdle et al., 2012).

However, this raises some theoretical issues. Because cognitive training techniques such as attention bias modification target the “attentional deployment” step of the emotion regulation model (Gross, 1998, 2014), which essentially cultivates the marginally effective strategy of distraction rather than that of “cognitive change”, dysfunctional appraisal processes are not targeted as they are in CBT. This is why such cognitive training paradigms might not act as a substitute for this form of psychotherapy: They might target important aspects of attention and memory, but they do not explicitly train the systems uniquely engaged during CBT. And, even if techniques were developed to train the systems underpinning cognitive restructuring and which act on maladaptive schemas and appraisals, they should probably still be used in combination with psychotherapy, since they would lack other important factors that contribute to mental health (e.g., interpersonal interactions that build the therapeutic alliance).

Whatever the optimal approach to cultivating mental health, the particular means by which exercise accomplishes its role is unclear. That physical activity facilitates the release of endorphins and dopamine (e.g., “runner's high”) is well-recognized, but also is the transient nature of these effects (see Dishman and O'Connor, 2009). Although these low-level neurobiological effects might be useful in boosting mood and decreasing anxiety, they might have little impact on sustained changes in mental health. Instead, any sustained effects might be due to changes in executive functions. In other words, positive affect from low-, state-level effects relating to exercise or otherwise is potentially not what drives current or future applications of cognitive strategies to downregulate negative emotion: Executive functions largely underpin this downregulation (see Gross, 2014). Therefore, profound changes in long-term mental health might be more closely related to the acute (functional) and chronic (structural) effects of exercise on the brain systems supporting executive functions. Much neuroimaging and neuropsychological research has localized executive functions in the frontal lobe, particularly the PFC (see Shallice and Cooper, 2011; Knight and Stuss, 2013 for reviews). For example, one executive function that might be critical to emotion regulation is the ability to regulate attention between stimulus-independent and –dependent information, a system which has been consistently localized within rostral PFC (BA10; see Burgess et al., 2007). Another important feature of executive functions that might be important is the general processing speed with these cognitive operations can be carried out.

However, what remains to be seen is whether psychopathological symptoms such as depressed mood relate to the acute effects of exercise on PFC functional activation during executive function tasks. The present work therefore aimed to investigate this question, as well as whether one's level of physical fitness, as measured by VO_2_peak, impacts the reactivity of the PFC to exercise. Because of the link between depressive symptoms and deficits in executive functions, and because exercise positively affects executive functions, there might also be a link between depressed people's neural reaction to exercise in the PFC. Thus, it was hypothesized that people with greater depressive symptoms might show weaker effects of exercise on the PFC, but that greater levels of cardiorespiratory fitness would show a stronger upregulation of the PFC as a function of exercise.

## Method

### Participants

Law enforcement officers were voluntarily recruited from UK-based forces and randomly allocated to an exercise group (*n* = 106; 73% male; age: 39 ± 9 years; weight 84.0 ± 19.7 kg; height 173.6 ± 20.2 cm), and a control group (*n* = 27; 97% male; age: 43 ± 6; weight 89.4 ± 18.6 kg; height 177.7 ± 9.4 cm). This sample of officers was part of a larger study looking at health and wellbeing in law enforcement and was not chosen for any specific characteristics (e.g., depression scores; fitness, age etc.) relating to this study. All participants provided written informed consent prior to participating in the study (Ethics number: 13985/004). All participants completed a physical activity readiness questionnaire (PAR-Q; Pescatello et al., 2014) to screen for eligibility to undergo maximal exercise testing and provided written informed consent prior to participating in the study. Participants were excluded from the study if they were not law enforcement officers, if they presented any injury or illness that prevented them from exercising to exhaustion, if they had a neurological condition or if they responded “yes” to any of the questions on the PAR-Q. Ethical approval was granted by the University College London Research Ethics Committee. An *a priori* power analysis of a medium effect size, with alpha set at (α = 0.05) (one-tailed) and beta 1–β (power) = 0.80, determined that *n* = 27 is adequate for within-subjects comparisons of exercise effects; however, a similar analysis suggested that between-subjects comparisons would require at least *n* = 46 for comparison with the experimental group.

### Procedure

**Figure 1:**
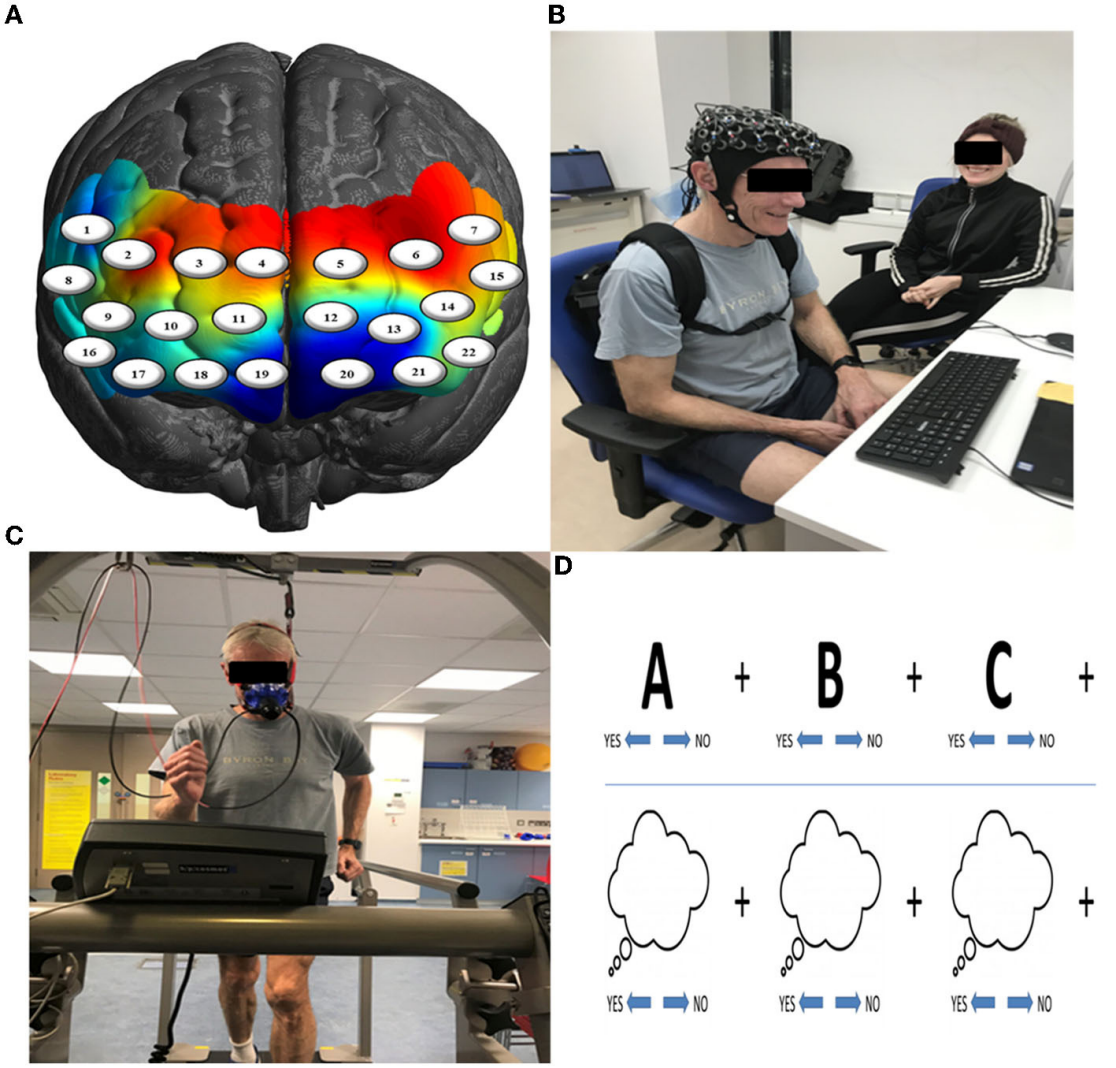
**(A)** Channel-specific locations of the 22-channel (eight sources & eight detectors) configuration overlaid onto a model brain mesh of the PFC. **(B)** Neurocognitive testing before and after exercise. **(C)** A 15-min bleep test of aerobic fitness to measure VO_2max_. **(D)** Example of trials of the SOT (upper row) and SIT (lower row) tasks.

Participants completed the Mood and Feelings Questionnaire (MFQ; Angold et al., 1995), a widely used self-report measure of depressive symptomology that has high internal reliability (Cronbach's alpha = 0.85). The experimental group completed neurocognitive testing before and after exercise, whilst the control group completed neurocognitive testing before and after resting (passively watching a television program), and then underwent exercise testing to assess their cardiorespiratory fitness. More specifically, participants completed a set of cognitive tasks on a computer screen, which were created in PsychToolbox, MATLAB (Mathworks, Natick, MA), to test executive functions such as inhibition and attention and, importantly, the general speed of processing across them (i.e., Speed). There were three blocks for inhibition that were adaptations of typical go/no go tasks (Donders, 1969), varying from low, moderate, to high inhibition. The low inhibition condition was a simple reaction time task. The moderate inhibition condition required participants to not respond to specific images (e.g., kittens). The high inhibition condition was the same as the moderate one, with the exception that a loud auditory noise would randomly occur (e.g., a gunshot). The last three blocks regarded mode of attending. These tasks required participants to respond in particular ways depending on whether they were attending to stimuli on the computer screen (i.e., stimulus-oriented thought: SOT) or independent of it (i.e., stimulus-independent thought: SIT) See Burgess et al. (2007) for review of these types of tasks. The first condition was SOT, the second was SIT, and the third was SIT with visual distractors (Figure 1).

The exercise group completed a VO_2_max test on a treadmill (h/p/cosmos, Nussdorf, Germany) using the Bruce protocol. The step protocol begins with a 3-min warm up, walking at 2.6 km/h with no incline, every 3 min thereafter the speed and incline of the treadmill increase, starting with a 10% incline at 2.7 km/h followed by incremental increases in both incline and speed every 3 min, pushing the participant to eventually running uphill, if their fitness allows. Throughout the test, participants were encouraged to continue exercising until volitional exhaustion, at which point the test was terminated. The treadmill was returned to level at walking pace, the participant was instructed to walk slowly (2.6 km/h) for 3 min to recover fully. Breath-by-breath gas analysis and heart rate were gathered through the Vyntus CPX Metabolic Cart (Vyaire Medical, Chicago, USA) throughout the test. The anaerobic threshold was determined through the v-slope method (Wasserman et al., 1994). VO_2_max (ml/kg/min) was determined as the highest recorded VO_2_ value. VO_2_max was identified as a true maximal value if the gas analysis showed a plateau in the VO_2_ values, respiratory exchange ratio (RER) exceeded 1.13, and heart rate max reached ~220-age. The exercise group included in the neuroimaging analysis reached true max (e.g., not quitting too early). After a recovery walk (3 min), they returned to the testing room, resulting in a ~10 min time delay between reaching maximal exertion and starting the second round of cognitive tests. To maximize data collection, the no-exercise group completed these protocols after the second round of cognitive tests.

### Physiological Measures

Participants were fitted with a continuous-wave functional near-infrared spectroscopy (fNIRS) system (LIGHTNIRS, Shimadzu Corp., Kyoto, Japan) to measure changes in hemodynamics, as well as with a physiological monitor to record heart and respiration rates (HR & RR, respectively). fNIRS signal acquisition used 16-fibers (22-channel configuration: 8 sources & 8 detectors), with a sampling rate of 13.33 Hz at three wavelengths of light (780, 805, and 830 nm). Digitization was based on a single subject due to the large sample size (*n* = 106) and need for rapid testing; the researchers were carefully trained to place the cap the same way for each participant. Anatomical locations of optodes in relation to standard head landmarks, including inion and top center (Cz) and left and right tragi, were determined using a Patriot 3D Digitizer (Polhemus, Colchester, VT). Montreal Neurological Institute (MNI) coordinates (Mazziotta et al., 2001) for each channel were obtained using NIRS-SPM software (Ye et al., 2009; https://www.nitrc.org/projects/nirs_spm/) with MATLAB (Mathworks, Natick, MA). The anatomical coverage of the channel configuration was over three bilateral ROIs (Table 1): rostral PFC (BA10), dorsolateral PFC (BA46/9), and ventrolateral PFC (BA44/ 45/47). These ROIs were specified *a priori* based on neuroimaging and neuropsychological research on frontal lobe functions (see Shallice and Cooper, 2011; Knight and Stuss, 2013, for reviews). ECG signals were continuously collected at 256 Hz using two Equivital “eq02+ LifeMonitors” (https://www.equivital.com/heart-rate-and-breathing-rate-monitor).

**Table 1:** Channels, coordinates, and anatomical regions.

**Channel #**	**Anatomical region**	**BA^**a**^**	**Coordinates^**b**^**
1	Right Superior Frontal Gyrus	9	53, 26, 30
2	Right Middle Frontal Gyrus	46	41, 49, 23
3	Right Rostral PFC	10	25, 62, 21
4	Right Rostral PFC	10	4, 65, 21
5	Left Rostral PFC	10	-19, 63, 21
6	Left Middle Frontal Gyrus	46	-37, 52, 22
7	Left Superior Frontal Gyrus	9	-51, 28, 29
8	Right Inferior Frontal Gyrus	44	60, 15, 16
9	Right Inferior Frontal Gyrus	45	51, 43, 7
10	Right Rostral PFC	10	36, 60, 5
11	Right Rostral PFC	10	17, 68, 5
12	Left Rostral PFC	10	-8, 68, 5
13	Left Rostral PFC	10	-32, 62, 4
14	Left Inferior Frontal Gyrus	45	-47, 46, 7
15	Left Inferior Frontal Gyrus	44	-58, 17, 17
16	Right Inferior Frontal Gyrus	47	54, 30, -6
17	Right Rostral PFC	10	45, 51, -8
18	Right Rostral PFC	10	28, 64, -7
19	Right Rostral PFC	10	3, 67, -7
20	Left Rostral PFC	10	-22, 66, -8
21	Left Rostral PFC	10	-41, 54, -7
22	Inferior Frontal Gyrus	47	-51, 36, -3

a *BA, Brodmann's Area*.

b *Coordinates are based on the MNI system and (–) indicates left hemisphere*.

Data analysis of the fNIRS data was completed in line with the quality control standards suggested by Yücel et al. (2021). Errors during data collection resulted in smaller sample sizes for the exercise (*n* = 92) and control (*n* = 18) groups. For example, participants were excluded if they did not have enough behavioral data to match the fNIRS data (i.e., for convolution of a stimulus design with a hemodynamic response function), or if their fNIRS data were too noisy or compromised in some way. The pre-processing of raw fNIRS signals was carried out on these data in accordance with Pinti et al. (2019). Namely, the raw voltage intensities were converted from.OMM format into.NIRS format. These data were then converted into optical density (OD) signals. Next, motion-artifact correction was completed using wavelet convolution (iqr = 1.5), with a differential pathlength factor (DPF) that is typically used for continuous-wave fNIRS [6, 6, 6]. These signals were temporally smoothed using a band-pass filter (FIR: order 1,000) [.01 .4 Hz]) to remove extracerebral, systemic effects. The cleaned OD signals were then converted into changes in concentrations of oxygenated hemoglobin (HbO_2_), deoxygenated hemoglobin (HbR), and total hemoglobin (HbO_2_ + HbR) using the modified Beer-Lampert Law (see Dirnagl and Villringer, 1997). Channels that were faulty or remained poor in signal-to-noise ratio were removed from the analysis.

HR (*n* = 56) and RR (*n* = 52) were included as additional parameters in the single-subject design matrices (see Tachtsidis and Scholkmann, 2016) to address variance due to physiological confounds in the predicted signals. Specifically, waveform analysis calculated RR from the ECG data (Charlton et al., 2016). HR was computed using the intervals between R-wave peaks of QRS complexes. Most of the physiological data were accounted for in the fNIRS sample (HR: 61%, RR: 57%), but not all. A comparison of the results with and without these physiological variables showed that accounting for the majority of physiological variance in the fNIRS sample was sufficient. Next, onsets and durations of the epochs of each trial of each block were extracted to generate a stimulus design for each participant, with which a canonical hemodynamic response function (HRF) was then convolved. The HR and RR parameters as well as the predicted HRFs (HbO_2_ and HbR) for each block were then down-sampled to 1 Hz using spline interpolation (Cohen, 2017) and a general-linear model analysis fitted these models to the observed data. The second-level analysis of the group data used a random-effects approach *via* summary statistics (Friston et al., 2007; Poldrack et al., 2011). The group effects of each HbO_2_ and HbR contrast for each channel were then projected onto a 3-D brain mesh *via* linear interpolation after false-discovery rate (FDR) correction (*q* < 0.05; Singh and Dan, 2006) was carried out. Finally, although both HbO_2_ and HbR signals were analyzed, the interpretation of the results was based on research suggesting that HbR signals are less affected by systemic confounds (Dravida et al., 2017), especially in fNIRS paradigms involving marked changes in arterial CO_2_ due to changes in respiration (e.g., exercise, speaking, etc.), because such changes alter the HbO_2_ signal to a greater degree than HbR in these cases (Scholkmann et al., 2013a,b).

## Results

**Figure 2:**
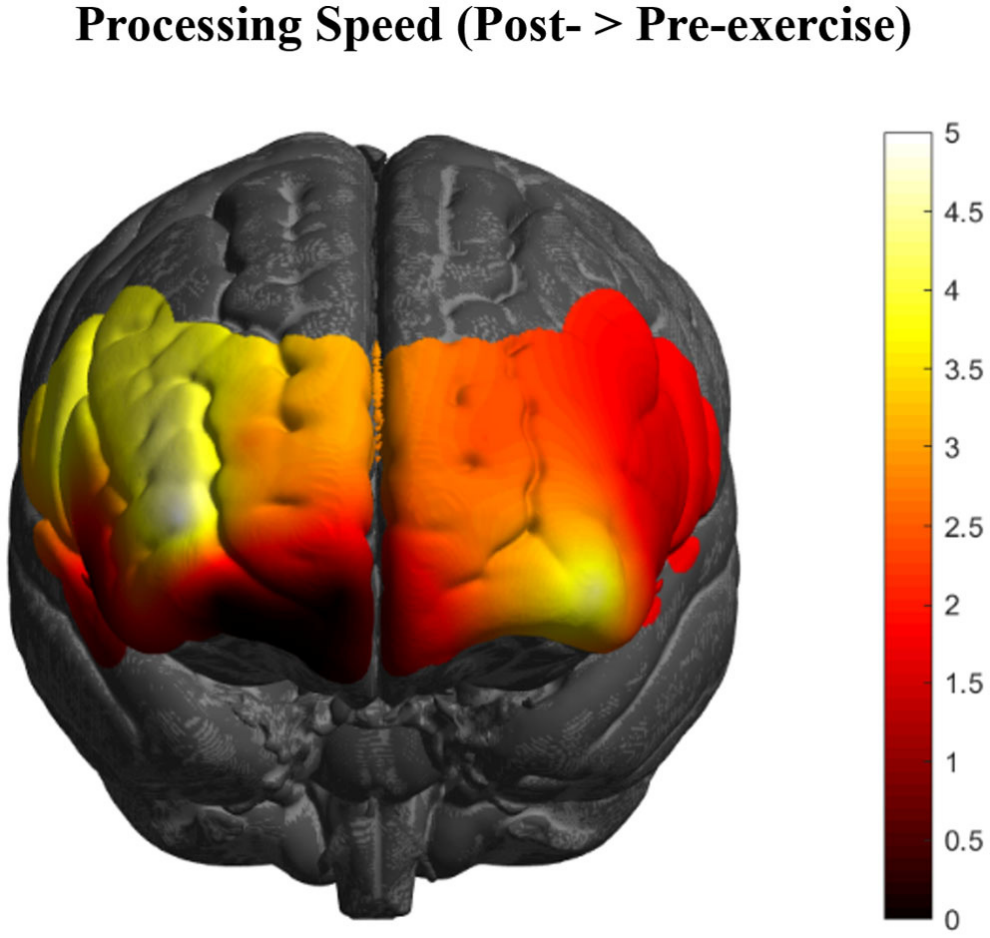
HbR changes in the PFC as an acute effect of exercise (*n* = 92) collapsed across all executive function conditions. Greatest activation changes are represented in bright yellow and white, with little to no effects represented in dark red and black, respectively (*t*-values of the images are scaled from 0 to 5+).

Changes in PFC activation from contrasting the pre- and post-exercise (post > pre) tasks of the experimental group (*n* = 92) showed bilateral activation changes in dorsolateral (BA46/9), ventrolateral (BA44/45), and rostral (BA10) PFC (Figure 2), with the greatest changes occurring in right BA10 and BA46 (*p'*s < 0.05, FDR corrected).

**Figure 3:**
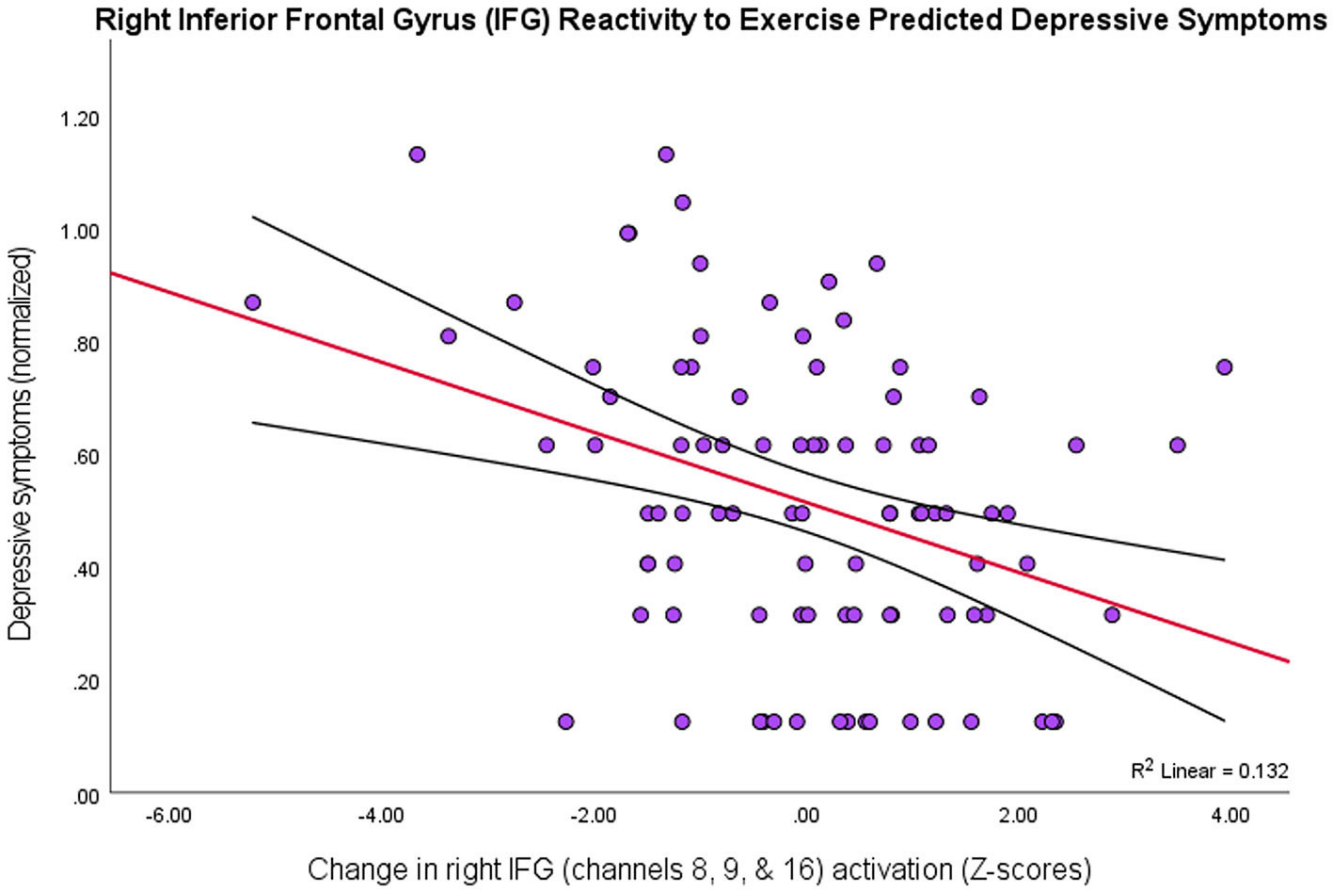
Higher depression scores on the MFQ (*y*-axis) predicted smaller changes in levels of activation in right IFG (BA44/45/47) as an effect of exercise (*r* = 0.36, *p* < 0.001, 95% CI [−0.07, −0.02]; 13% variance explained). The red line depicts the regression line, with black lines as confidence intervals.

To investigate whether the effects observed in these PFC subregions varied depending on level of depression symptomology (MFQ scores; *M* = 4.04, *SD* = 4.25), a multiple linear regression analysis resulted in three channels (8, 9, & 16) that explained the most variance in depression scores, *R*^2^ = 0.14, *F*_(2, 89)_ = 4.59, *p* = 0.005. Together, these channels form a spatial cluster approximately over right inferior frontal gyrus (IFG: BA44/45/47, respectively), so the values for these channels were transformed into *Z*-scores and summed to create an “IFG” variable. Although the IFG data were normally distributed, a Shapiro-Wilk test showed that the depression scores were not normally distributed, *W* =.80, *p* < 0.001 (Skewness = 2.01, Kurtosis = 5.37). Therefore, the IFG effects were regressed onto a normalized distribution of depression scores (Skewness = 0.21, Kurtosis = −0.64), yielding a more statistically robust model, *R*^2^ = 0.13, *F*_(1, 89)_ = 12.91, *p* < 0.001, of right IFG as a predictor variable, *t*_(89)_ = −3.59, *p* < 0.001, 95% CI [−0.07, −0.02] (Figure 3).

Thus, there was a negative relationship between depression scores and the PFC activity that was due to exercise-elicited, processing-speed changes, particularly in right inferior frontal gyrus (BA44/45/47). That is, people with greater symptoms of depression showed lower levels of PFC activation across all tasks after exercise.

**Figure 4:**
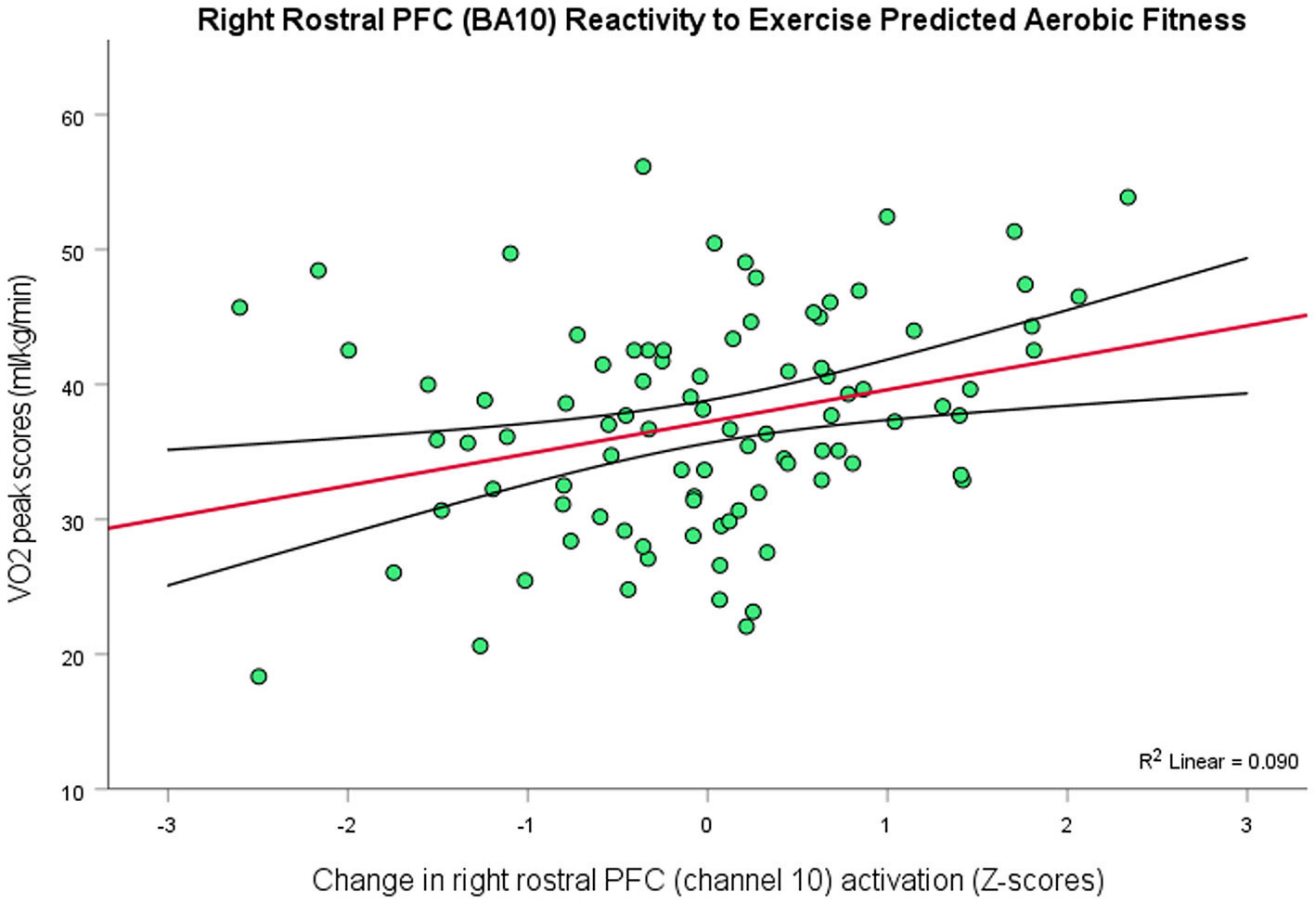
Higher levels of aerobic fitness (*y*-axis) predicted greater changes in the level of activation in right rostral PFC (BA10) as an effect of exercise (*r* = 0.30, *p* = 0.004, 95% CI [0.79, 3.97]; 9% variance explained). The red line depicts the regression line, with black lines as confidence intervals.

Following the same procedures for VO_2_peak scores (*M* = 37.25, *SD* = 8.24) to investigate whether physical fitness predicted the PFC effects of exercise, since these data were also not normally distributed, *W* = 0.94, *p* < 0.001 (Skewness = −1.14, Kurtosis = 3.97), a one channel model, *R*^2^ = 0.09, *F*_(1, 89)_ = 8.81, *p* = 0.004, 95% CI [0.79, 3.97], containing channel 10 (right rostral PFC; BA10) emerged as a significant predictor of a normalized distribution of VO_2_peak scores (Figure 4). So, there was a positive relationship between VO_2_peak and exercise-elicited changes in PFC activity in right rostral PFC (BA10), in that people with greater aerobic fitness showed greater levels of activation in this region after exercise. However, there was no association between VO_2_peak and depressive scores, *r*_(90)_ = −0.05, *p* > 0.05.

## Discussion

Our results show some relationships between symptoms of depression and exercise-elicited changes in PFC activity, as well as between this activity and level of fitness. Specifically, there was a negative relationship between symptom severity and activity in right inferior frontal gyrus (BA44/45/47), in that greater activation changes in these subregions were associated with lower depression scores. Right inferior frontal gyrus (i.e., ventrolateral PFC) has been consistently implicated in the controlled downregulation of negative emotion (Zilverstand et al., 2017) and is an area that is relatively hypoactive during emotion regulation in depressed populations (Rive et al., 2013). So, the evidenced negative relationship is consistent with the idea that this area of the PFC is underactive during executive function tasks in people with depressive symptoms (see Wang et al., 2008). Although this relationship was not found in rostral PFC, it does not mean that there is no link between this subregion and mood in relation to exercise. A stronger relationship might be evidenced should alternative self-report measures be used that index not only mood and negative affect but also the dysfunctional cognitions that underpin them (e.g., the shorter Attitudes and Belief Scale 2; DiGiuseppe et al., 2021), as well as people's tendency to downregulate negative affect and mood from a 'top-down' approach (i.e., reappraisal), such as with the Emotion Regulation Questionnaire (Gross and John, 2003). Such measure might mediate the relationship between PFC reactivity and depressive symptoms.

In addition, there was a positive relationship between aerobic fitness (VO_2_peak) and a different area of right PFC (BA10), in that greater fitness was predicted greater activation changes in right rostral PFC. While decreased activation of the PFC appears to be the rule in depression (see Hariri, 2015), there are some exceptions: For example, one study recently showed that pessimistic future-thinking in people with major depressive disorder exhibited greater activity in right rostral PFC compared to healthy individuals (Katayama et al., 2019). Given the cognitive operations for which this brain region is largely functionally specialized (i.e., regulating thought that is dependent or independent of the external world; Burgess et al., 2007), it makes sense that dysfunctional, prospective appraisals would be supported by the biasing of stimulus-independent thought—that, here, there would be hyperactivity in depressed individuals. Equally supported by this orientation of attention are the hypothesized cognitive operations of “recogitation” (Crum, 2021b)—executive functions in which depressed people appear to have deficits—and, therefore, hypo-activity in BA10 might be present during their implementation. Thus, rostral PFC is potentially as much involved in the pathogenesis of psychopathological symptoms as it is in facilitating the cognitive change mechanisms that ameliorate them. So, it is plausible that the relative hypo- or hyper-activation of this brain region might depend on the particular type of executive function task in which depressed individuals engage. For example, collapsing across different executive function tasks might result in BA10 activation that explains little variability in depression, as was the case in the present study, but that the exercise effect in this PFC subregion was sensitive to how aerobically fit people were might suggest an important role of fitness in treating depression. That is, it is plausible that physical fitness works to correct issues of abnormal activity in rostral PFC (e.g., Saanijoki et al., 2022). Future investigations might further elucidate this possibility, but the present findings represent a starting point for highlighting the potential interrelations between the PFC, physical activity, and depressive symptoms, as well as between physical activity, rostral PFC, and aerobic fitness.

A limitation of the present work was that long-term changes in cognition, behavior, brain, fitness, and mood were not measured. This constrains the type of inferences that can be made about their interrelations, so future research investigating the chronic effects of exercise on the brain, particularly the PFC, might examine how functional changes in ventrolateral and rostral PFC related to executive function tasks vary with changes in psychopathological symptoms. Another possibility for future research is to examine how much and what kind of physical activity are sufficient to facilitate marked mental health changes. For example, are a few acute bouts of exercise enough to cultivate these changes or do overall fitness levels need to be increased, which is achieved through more repeated, habitual activity? Another limitation was that post-test measures in mood were not taken, so it was not possible to assess the acute effects of exercise on mood. However, this is not a significant limitation given the current theoretical presuppositions about how exercise improves mood over time. Namely, although physical activity tends to indeed improve positive affect due to low-level neurobiological changes (e.g., runners' high), such effects are typically transient (Dishman and O'Connor, 2009) and do not constitute mood. Therefore, it is perhaps to the potential effects of exercise on top-down cognitive strategies within the PFC to which researchers ought to look for explaining lasting improvements to mood and the ability to downregulate negative emotion.

Such mediating effects of the PFC might explain the lack of a negative relationship between physical fitness and depressive symptoms and, more broadly, why there are many people who are depressed despite being physically fit. Indeed, the relationship between increases in aerobic fitness and decreases in depressive symptoms is relatively weak (Martinsen et al., 1989). So, it is perhaps not enough to be fit: The benefits of exercising on cerebral oxygenation in the brain ought also to be taken advantage of in order to augment the facilitation of regulation strategies (i.e., the mind needs to also play an active role in cultivating mental health). Interestingly, improvements to executive functions during and after exercise are optimized to the extent that these functions are a part of the task demands of a physical activity (e.g., hunting was a cognitively demanding exercise task for early humans; see Raichlen and Alexander, 2017). Therefore, it could be the case that the influence of exercise on decreasing psychopathological symptoms is optimized to the extent that an exercise also requires of the individual the executive functions that are integral to the cognitive change strategies that drive future efforts to downregulate the onset of negative affect. This raises the interesting question of whether the physical activities of extremely fit, yet depressed individuals are not sufficiently enriched, cognitively. At this stage, however, these must remain speculations, but the results presented here are consistent with the possibility that there may be a unifying neural mechanism that links the effects of physical activity upon the brain, consequent changes in cognitive processing, and depression-like symptoms. The potential clinical implications for the role of physical activity and fitness in treatment seem clear, so it would be highly valuable to investigate these results in a clinical population.

## Data Availability Statement

The raw data supporting the conclusions of this article will be made available by the authors, without undue reservation.

## Ethics Statement

The studies involving human participants were reviewed and approved by University College London Research Ethics Committee. The patients/participants provided their written informed consent to participate in this study.

## Author Contributions

JC, FR, and PB designed the experiment. JC, GH, SF, and EC collected the data. JC analyzed the data and wrote the manuscript. FR, GH, SF, EC, UH, IJ, MH, JH, AH, IT, and PB reviewed and suggested revisions to it. All authors contributed to the article and approved the submitted version.

## Conflict of Interest

The authors declare that the research was conducted in the absence of any commercial or financial relationships that could be construed as a potential conflict of interest.

## Publisher's Note

All claims expressed in this article are solely those of the authors and do not necessarily represent those of their affiliated organizations, or those of the publisher, the editors and the reviewers. Any product that may be evaluated in this article, or claim that may be made by its manufacturer, is not guaranteed or endorsed by the publisher.
